# Positive psychosocial factors and the development of symptoms of depression and posttraumatic stress symptoms following acute myocardial infarction

**DOI:** 10.3389/fpsyg.2023.1302699

**Published:** 2023-12-04

**Authors:** Claudia Zuccarella-Hackl, Lucia Jimenez-Gonzalo, Roland von Känel, Mary Princip, Lena Jellestad, Rebecca E. Langraf-Meister, Hansjörg Znoj, Jean-Paul Schmid, Jürgen Barth, Ulrich Schnyder, Katharina Ledermann

**Affiliations:** ^1^Department of Consultation-Liaison Psychiatry and Psychosomatic Medicine, University Hospital Zurich, University of Zurich, Zurich, Switzerland; ^2^University Rey Juan Carlos of Madrid, Madrid, Spain; ^3^Clienia Schlössli AG, Zurich, Switzerland; ^4^Department of Health Psychology and Behavioral Medicine, University of Bern, Bern, Switzerland; ^5^Department of Cardiology, Clinic Gais, Gais, Switzerland; ^6^Institute for Complementary and Integrative Medicine, University Hospital Zurich, University of Zurich, Zurich, Switzerland; ^7^University of Zurich, Zurich, Switzerland; ^8^Department of Psychology, University of Fribourg, Fribourg, Switzerland

**Keywords:** positive psychosocial factors, depressive symptoms, posttraumatic stress symptoms, acute myocardial infarction, cluster analysis

## Abstract

**Introduction:**

Acute myocardial infarction (MI) is a potentially fatal condition, leading to high psychological distress and possibly resulting in the development of depressive symptoms and posttraumatic stress symptoms (PTSS). The aim of this study was to investigate the association of clusters of positive psychosocial factors (resilience, task-oriented coping, positive affect and social support) with both MI-induced depressive symptoms and PTSS, independent of demographic factors.

**Methods:**

We investigated 154 consecutive patients with MI, 3 and 12 months after hospital discharge. All patients completed the short version of the German Resilience Scale, the Coping Inventory for Stressful Situations (CISS), the Enriched Social Support Inventory (ESSI) and the Global Mood Scale (GMS). The level of interviewer-rated MI-induced posttraumatic stress disorder (PTSD) symptoms at 3- and 12-months follow-up was evaluated through the Clinician-Administered PTSD Scale (CAPS). Depressive symptoms were assessed at 3- and 12-month follow-up with the Beck Depression Inventory (BDI-II).

**Results:**

Three different clusters were revealed: (1) lonely cluster: lowest social support, resilience and average task-oriented coping and positive affect; (2) low risk cluster: highest resilience, task-oriented coping, positive affect and social support; (3) avoidant cluster: lowest task-oriented coping, positive affect, average resilience and social support. The clusters differed in depressive symptoms at 3 months (*F* = 5.10; *p* < 0.01) and 12 months follow-up (*F* = 7.56; *p* < 0.01). Cluster differences in PTSS were significant at 3 months (*F* = 4.78, *p* < 0.05) and 12 months (*F* = 5.57, *p* < 0.01) follow-up. Differences in PTSS subscales were found for avoidance (*F* = 4.8, *p* < 0.05) and hyperarousal (*F* = 5.63, *p* < 0.05), but not re-experiencing, at 3 months follow-up. At 12 months follow-up, cluster differences were significant for re-experiencing (*F* = 6.44, *p* < 0.01) and avoidance (*F* = 4.02, *p* < 0.05) but not hyperarousal.

**Discussion:**

The present study contributes to a better understanding of the relationships among different positive psychosocial factors, depressive symptoms and PTSS following acute MI. Future interventions may benefit from taking into account positive psychosocial factors to potentially reduce patients’ depressive symptoms and PTSS after MI.

## 1 Introduction

A myocardial infarction (MI) is a potentially traumatic event that may result in post-traumatic stress disorder (PTSD; Kutz et al., 1994; Jacquet-Smailovic et al., 2021). Studies have shown that in the aftermath of an acute coronary syndrome (ACS), 12% of patients develop clinically relevant posttraumatic stress symptoms (PTSS) (Edmondson et al., 2012). PTSD is defined as a composite of various psychological, physiological, and behavioral symptoms including intrusive thoughts, avoidance, negative alterations in cognitions and mood, hyperarousal and increased stress reactivity (American Psychiatric Association, 2013). PTSS after MI, which are underdiagnosed and often go unrecognized by health care providers, can adversely affect recovery. Specifically, MI-induced PTSS may have a negative impact on patients’ overall and cardiovascular health (Ginzburg and Ein-Dor, 2011). Several pre-traumatic risk factors for the development of MI-induced PTSS have been identified, including a history of PTSD (Guay et al., 2002), cardiovascular disease (Kutz et al., 1994) and substance abuse (DeVaul, 1999).

In addition to PTSS, accumulated evidence has consistently shown that depression is one of the most common psychological reactions in the aftermath of MI which may not only lead to impaired long-term quality of life, but also cause increased mortality among patients with MI (Bush et al., 2001; Hosseini et al., 2014). Pooled prevalence rates of depression among patients with MI in ten different countries have been reported as high as 28.70% (Feng et al., 2019). Depression in patients with MI can lead to delayed recovery, increased risk of complications, reduced medication adherence, and a poorer quality of life. Depressed individuals may engage in unhealthy behaviors, suffer from psychological distress and become socially isolated, which can exacerbate the physical and emotional burdens of MI (Hosseini et al., 2014).

Although the impact of MI on psychopathological problems (e.g., depression, distress, anxiety) has gained increasing attention, much less is known about the presence and role of protective psychosocial factors, which may mitigate the development of PTSS following MI. This perspective is also important because there is substantial evidence that positive psychological states are associated with better health outcomes and reduced morbidity (Lyubomirsky et al., 2005; Pressman and Cohen, 2005), including in cardiovascular disease (Sin, 2016). Positive well-being may serve as a modifiable protective psychosocial factor that could reduce the burden of MI through its potential influences on lifestyle behaviors and MI-related biomarkers (Cipollone et al., 2004). Previous literature has considered social support and individual variables such as positive affect (Lee et al., 2013; Martínez-Martí and Ruch, 2017), suggesting that both internal and external resources would influence outcomes such as depression or PTSD. Aspects of positive psychological well-being have been identified as positive health assets, and they have been linked to improved outcomes related to cardiovascular disease (Boehm and Kubzansky, 2012; Kubzansky et al., 2018). Therefore, we examined to what extent positive psychosocial factors like resilience, coping, positive affect and social support might potentially reduce the development of depressive symptoms and PTSS after MI.

Resilience is a concept widely defined as the dynamic capacity to adapt effectively in the presence of adversity, trauma, or significant threats (Southwick and Charney, 2018). From a trait-oriented perspective, resilience is considered an inherent personal characteristic that empowers individuals to skillfully confront challenge, adapt, and flourish (Hu et al., 2015). Proponents of this view regard resilience as a stable personality trait that serves as a protective buffer, mitigating the adverse effects of adversity and traumatic experiences (Ong et al., 2006). Conversely, an outcome-oriented viewpoint perceives resilience as a behavioral result or a functional outcome, capable of overcoming traumatic events and assisting individuals in their recovery from adversity (Harvey and Delfabbro, 2004). On the other hand, the process oriented approach conceptualizes resilience as a dynamic ongoing process in which individuals actively engage to adapt and swiftly recover from significant adversities (Luthar et al., 2000; Fergus and Zimmerman, 2005).

Several studies have shown negative association of trait resilience on the development of PTSD (Daniels et al., 2012). Meister et al. (2015) demonstrated that a high trait resilience score correlates negatively with acute stress during MI and can thus be considered a potential protective factor for the development of PTSS after MI. In addition, resilience has been associated with better processing of negative emotions (Kirchner et al., 2022), thereby reducing the likelihood of MI-induced PTSS. Lastly, trait resilience has been shown to be positively associated with quality of life 1 year after MI (Kirchberger et al., 2020).

The literature on post-MI adjustment emphasizes the importance of individual coping strategies. For example, Chung et al. (2008) found that people who used emotion-focused or avoidant coping strategies were more likely to report PTSS and other symptoms of psychological distress after MI. Several other studies confirmed this finding (Ayers et al., 2009; Marke and Bennett, 2013; Burnos and Wrzosek, 2022). On the other hand, high task-oriented coping has been associated with lower depression scores and more favorable illness attitude in patients with ACS (Wrześniewski et al., 1994; Messerli-Bürgy et al., 2015).

In contrast to the above literature, the potential influence of positive affect on the development of PTSS after MI has largely been neglected. Positive affect refers to the tendency to experience pleasurable emotions, including joy, happiness, excitement, enthusiasm, and contentment (Pressman and Cohen, 2005) and is not merely the opposite of negative affect, as both types of affect can be present simultaneously (Larsen et al., 2001). High levels of positive affect have been shown to reduce the risk of mortality in cardiovascular populations (Scherer and Herrmann-Lingen, 2009), cardiovascular risk factors and secondary cardiovascular events (Sin, 2016). Positive well-being is also linked to better immune, neuroendocrine, and cardiovascular functioning, in addition to reduced stress reactivity and adaptive coping skills in cardiac patients (Sin, 2016; Zuccarella-Hackl et al., 2023).

Finally, various studies have demonstrated the protective effect of social support on the development of PTSS after MI (Bennett and Brooke, 1999; Pedersen et al., 2004; Marke and Bennet, 2013). A recent study showed that an acute psychological stress response correlated negatively with social support in younger MI patients (Wu et al., 2022).

In this context, cluster analysis is a promising method for identifying and describing subgroups of individuals based on similarities across multiple dimensions, such as positive psychosocial factors. By organizing large amount of information coming from different measurements, cluster analysis allows to group a heterogeneous sample into relatively homogeneous groups. This method has previously been used to study different coping profiles and its relationship with health behaviors (Doron et al., 2015). However, as far as we are aware, it has not been used in order to study positive psychosocial factors and its relationship with patients’ distress after an MI.

In summary, resilience, task-oriented coping, positive affect and social support appear to be important factors for both psychological well-being and physical health. However, to our knowledge, there are no investigations of these factors in conjunction and their influence on depression and PTSS development after MI. The aim of this study was to investigate, through cluster analysis, the association between clusters of positive psychosocial variables (resilience, task-oriented coping, positive affect and social support) with MI-induced depressive symptoms and PTSS, independent of patient profile. Additionally, secondary analyses were performed to explore differences in each PTSD subscale (re-experiencing, avoidant, and hyperarousal) related to the profile of positive psychosocial factors.

## 2 Materials and methods

### 2.1 Participants

Participants from the present study were a subsample from the Myocardial Infarction-Stress Prevention Intervention (MI-SPRINT) randomized controlled trial (RCT). The aim of the MI-SPRINT project was to examine whether psychological counselling shortly after a hospital admission due to an MI event could prevent PTSS related to such event (von Känel et al., 2018). For the present study, information was available from 154 participants at baseline and at 3-month follow-up assessment, and from 104 participants at 12-month follow-up assessment.

Inclusion criteria to take part in the study were: (1) to be at least 18 years old; (2) to have had a confirmed acute ST-elevation myocardial infarction (STEMI) or non-STEMI; (2) to have stable circulatory conditions (i.e., no signs of cardiogenic shock, such as lividness, uneasiness, cold sweats, heart rates higher than 100/min, or systolic blood pressure lower than 100 mmHg); (3) to have experienced high levels of distress during the MI episode. Distress levels was determined by scores ≥5 on a numeric rating scale of 0–10 for “pain intensity (during MI),” “fear of dying (till admission to the coronary care unit)” and/or “feelings of worry and helplessness (after being advised about having MI).” Exclusion criteria were: (1) patients who had emergency coronary artery bypass grafting; (2) patients with a severe illness entailing a high risk of dying within a year; (3) disoriented or with cognitive impairment; (4) with current clinical depression or a history of severe clinical depression; (5) suicidal thoughts in the previous 2 weeks; (5) insufficient knowledge of German; and (6) already enrolled in another clinical trial.

### 2.2 Procedure

The recruitment and intervention information has been outlined elsewhere (Meister et al., 2013; von Känel et al., 2018, 2021). Participants were recruited between January 2013 and December 2015, and they were referred to the Cardiology Department at Bern University Hospital in Berne, Switzerland. Participants were assessed at three different time points. Firstly, they were asked to complete a battery of validated questionnaires within 48 h of experiencing an MI (baseline measures). Participants were later contacted again 3 months after hospital admission (3-months follow-up assessment) and 12 months after hospital admission (12-months follow-up assessment). The study was carried out according to the Good Clinical Practice Guidelines and the Declaration of Helsinki, meaning voluntary participation, informed consent, anonymity, confidentiality, potential for harm, and results communication. Furthermore, the study was registered with ClinicalTrials.gov (NCT01781247), approved by the State of Bern’s ethic committee (KEK No. 170/12) and independently monitored by the Clinical Trials Unit at the University of Bern. All participants provided written informed consent before participating in the study and did not receive any form of monetary compensation.

### 2.3 Measures

#### 2.3.1 Admission measures

##### 2.3.1.1 Demographic and medical variables

Information was gathered regarding participants’ age and gender, as well as medical variables including assessment of acute STEMI or non-STEMI, evaluation of recurrent myocardial infarction, body mass index (BMI), hypertension, hypercholesterolemia, diabetes mellitus, systolic and diastolic blood pressure.

##### 2.3.1.2 Social support

The Enriched Social Support Inventory (ESSI; Mitchell et al., 2003) was used to assess social support. The ESSI measures the construct of social support through a 7-item scale regarding three dimensions, structural support (e.g., “Are you currently married or living with a partner?”), instrumental support (e.g.: “Do you have someone to help you with daily duties and work?”), and emotional support (e.g.: “Do you have someone who will listen to you when you feel the need to talk?”). Items are rated on a five-point Likert scale (0 = “never” to 4 = “always”). A high ESSI total score reflects a high level of social support. For the present study, the Cronbach’s α was 0.89.

#### 2.3.2 3-month follow-up measures

To minimize patient burden during hospital admission, the assessment of positive affect, resilience and coping were included in the 3-month follow-up interview.

##### 2.3.2.1 Positive affect

The positive affect subscale from the German version of the Global Mood Scale (GMS) was used to assess positive affect (Denollet, 1993). It consists of 10 items (e.g., cheerful, lively, dynamic) rated on a 5-point Likert scale (0 = not at all, 4 = extremely). For the present study, the Cronbach’s α was 0.88.

##### 2.3.2.2 Resilience

The short form of the validated German version (Schumacher et al., 2005) of the Resilience Scale (RS-11) by Wagnild and Young (1993) was used in order to assess resilience in the participants. The RS-11 consists of 11 items (e.g., “I often find something to laugh about”) that evaluate the construct of resilience as a single dimension using a seven-point Likert scale from “1 = I do not agree” to “7 = completely agree.” A higher total score on the RS-11 indicates a greater level of resilience. For the present study, the Cronbach’s α was 0.92.

##### 2.3.2.3 Coping

In order to assess coping styles, the German shortened version of the Coping Inventory for Stressful Situations (CISS) developed by Kälin (1995) was used. Participants rated 24 items on a 5-point Likert Scale ranging from 1 = “not at all” to 5 = “very much.” The questionnaire evaluated three different coping styles: task-oriented coping, emotion-focused coping, and avoidance-oriented coping. This study focused specifically on the task-oriented coping subscale (e.g., “I think about how I have solved similar problems”), which showed an internal consistency of Cronbach’s α of 0.87.

#### 2.3.3 Dependent variables

##### 2.3.3.1 Depressive symptoms

The German version (Kühner et al., 2007) of the Beck Depression Inventory second edition (BDI-II) (Ahrari et al., 2013) was used. To reduce the number of questions only the cognitive symptoms subscale was utilized. This particular subscale comprises 13 items (e.g., “sadness,” “feeling like a failure”) that were ranked on a Likert scale ranging from 0 to 3, resulting in a total score range of 0–39. Participants were asked to complete the BDI-II both at the 3-month follow-up interview and at the 12-month follow-up interview. Previous studies have applied the BDI-II cognitive subscale to patients with medical conditions such as MI (Poole et al., 2009). The internal consistency of Cronbach’s α was 0.71 in our sample.

##### 2.3.3.2 Post-traumatic stress symptoms

The severity of MI-induced PTSS was measured using the Clinician-Administered PTSD scale (CAPS), validated for German speaking samples (Schnyder and Moergeli, 2002). Psychology doctoral and medical master students received education and supervision from senior clinical psychotherapists in conducting the CAPS interview. Prior to independently conducting the CAPS interview, the interviewers completed a comprehensive 2-day training program. The frequency and severity of each of the 17 PTSS listed in the Diagnostic and Statistical Manual of Mental Disorders (DSM-IV) over the preceding months were evaluated. Ratings ranged from 0 (never) to 4 (nearly always) to produce a total severity score of PTSS related to the MI event ranging from 0 to 136. When frequency was at least 1 point and intensity was at least 2 points, a symptom was endorsed. The reexperiencing cluster required one of five symptoms, the avoidance cluster required three of seven symptoms, and the hyperarousal cluster required two of five symptoms. Participants were assessed on their PTSS both at the 3-month follow-up interview and at the 12-month follow-up interview. Cronbach’s α was 0.79 at 3-month and 0.72 at 12-month follow-up for CAPS total score in our sample.

### 2.4 Data analysis

An Euclidean distance matrix and a hierarchical cluster analysis was performed using Ward’s method in order to identify homogeneous groups in the sample based on participants’ scores on the assessed variables. As a previous step, missing data were replaced using Expectation Maximization. Also, with the aim of each variable to contribute equally to the distance measure in cluster analysis, the considered variables (resilience, task-oriented coping, positive affect, and social support) were converted to standardized (Z) scores. That way, we aimed to ensure comparability and prevent scale effects when analyzing distance measures in cluster analysis. An initial exploratory suggested a 3-cluster structure. Thus, 3 clusters were selected based on the information contained in the dendogram, illustrating the existence of possible natural clusters (please refer to Supplementary material). As a second step, one-way ANOVA analysis were carried out to identify differences among clusters in the assessed variables (depressive symptoms and PTSS). Additionally, factor analysis was carried out to identify the factor structure of the positive psychosocial variables, and differences between cluster groups in sociodemographic and medical variables were also analyzed through ANOVAs and chi-square tests. This information can be found in the Supplementary material. All analyses were carried out using the IBM SPSS program (version 22.0) with a level of significance set at *p* < 0.05. Means, standard deviation, ranges and frequencies are shown as descriptive data. As a final step, effect size measures for one-way ANOVAs were calculated as eta-squared measures.

## 3 Results

Participant’s demographic characteristics of the sample are given in Table 1. Most of the sample was composed of male participants with an average age of 59 years, most of the MI episodes were first-time STEMI MI.

**Table 1:** Characteristics of the sample at 3-month follow-up (*n* = 154) and at 12-month follow-up (*n* = 104).

	Overall sample (*n* = 154)		Cluster 1 (*n* = 50)		Cluster 2 (*n* = 50)		Cluster 3 (*n* = 54)	
	Mean (SD)/Percentages (*N*)	Observed range	Mean (SD)/Percentages (*N*)	Observed range	Mean (SD)/Percentages (*N*)	Observed range	Mean (SD)/Percentages (*N*)	Observed range
**Demographics**								
Gender, male %	84.4 (130)		76.0 (38)		86.0 (43)		90.70 (49)	
Age	58.96 (10.40)	33–84	58.0 (10.54)	37–84	59.68 (10.86)	33–78	59.17 (9.97)	39–84
**Medical variables**								
STEMI, %	71.4 (110)		62.0 (31)		80.0 (40)		72.2 (39)	
Recurrent MI, %	8.4 (13)		10.0 (5)		4.0 (2)		11.1 (6)	
Body Mass Index	27.83 (4.67)	15.90–42.97	27.63 (4.84)	16–40	27.53 (4.15)	19–39	28.31 (4.99)	20–43
Hypertension, %	48.7 (75)		42.0 (21)		50.0 (25)		53.7 (29)	
Hypercholesterolemia, %	44.2 (68)		36.0 (18)		48.0 (24)		48.1 (26)	
Diabetes, %	12.3 (19)		12.0 (6)		6.0 (3)		18.5 (10)	
Systolic blood pressure, mm Hg	137.04 (28.39)	95–262	136.49 (30.56)	95–262	141.03 (29.83)	96–200	133.71 (24.81)	100–185
Diastolic blood pressure, mm Hg	79.74 (15.86)	43–140	77.8 (17.10)	48–140	79.24 (18.17)	43–115	82.14 (11.89)	57–110
**Pychometric scales, scores**								
Resilience	61.23 (11.11)	20–77	56.32 (10.46)	20–76	69.95 (4.50)	59–77	57.70 (11.38)	25–73
Task-oriented coping	29.86 (5.62)	8–40	29.64 (4.60)	19–38	34.44 (3.90)	24–40	25.84 (4.60)	8–32
Positive affect	18.66 (6.73)	3–36	17.92 (5.71)	5–34	23.08 (6.38)	7–36	15.25 (5.66)	3–28
Social support	20.49 (4.04)	7–25	15.94 (3.24)	7–23	22.79 (2.11)	18–25	22.58 (2.05)	17–25
Depressive symptoms at 3-month follow-up	5.51 (5.16)	0–31	6.41 (4.69)	0–25	3.49 (3.65)	0–16	6.46 (6.17)	0–31
Depressive symptoms at 12-month follow-up	5.27 (4.65)	0–27	7.28 (4.28)	0–17	3.03 (3.18)	0–10	5.70 (5.08)	0–27
Post-traumatic Stress Symptoms at 3-month follow-up	10.64 (10.52)	0–54	12.44 (9.80)	0–49	6.94 (8.19)	0–47	12.39 (12.23)	0–54
Reexperiencing symptoms at 3-month follow-up	2.73 (4.12)	0–21	3.04 (4.13)	0–21	2.16 (4.07)	0–21	2.98 (4.19)	0–15
Avoidance symptoms at 3-month follow-up	2.94 (4.34)	0–25	3.18 (3.62)	0–19	1.36 (2.13)	0–10	4.17 (5.84)	0–25
Hyperarousal symptoms at 3-month follow-up	4.94 (4.38)	0–19	6.18 (4.74)	0–19	3.38 (3.39)	0–16	5.24 (4.48)	0–17
Post-traumatic Stress Symptoms at 12-month follow-up	9.50 (8.74)	0–52	13.32 (10.80)	0–52	6.41 (5.82)	0–25	9.15 (8.17)	0–31
Reexperiencing symptoms at 12-month follow-up	2.07 (3.14)	0–18	3.68 (4.37)	0–18	1.38 (2.50)	0–8	1.38 (1.77)	0–6
Avoidance symptoms at 12-month follow-up	2.96 (4.11)	0–18	4.06 (4.97)	0–18	1.41 (1.96)	0–6	3.44 (4.41)	0–18
Hyperarousal symptoms at 12-month follow-up	4.38 (3.78)	0–16	5.26 (3.65)	0–16	3.62 (3.76)	0–14	4.33 (3.83)	0.15

### 3.1 Cluster analysis

As can be seen in Supplementary Figure S1, initial cluster analysis proposed the existence of 3 different clusters. Table 2 depicts means and standard deviations of the variables considered for each of the 3 clusters, along with comparison (ANOVA) between the groups in such variables (*F* value and *post-hoc* comparisons).

**Table 2:** Means, standard deviations and differences between means in the assessed variables for each cluster.

Cluster	N	Resilience mean (SD)	Task oriented coping mean (SD)	Positive affect mean (SD)	Social support mean (SD)
1	50	53.32 (10.46)	29.64 (4.60)	17.92 (5.71)	15.94 (3.24)
2	50	69.95 (4.50)	34.44 (3.90)	23.08 (6.38)	22.79 (2.11)
3	54	57.70 (11.38)	25.84 (4.60)	15.25 (5.66)	22.58 (2.05)
Total	154	61.23 (11.11)	29.86 (5.62)	18.66 (6.73)	20.49 (4.04)
Mean differences (*F* values)		32.45***	49.94***	23.28***	121.32***
Effect size		0.30	0.40	0.24	0.62
*Post-hoc* analysis		1 and 3 < 2***	3 < 1 and 2*** 1 and 3 < 2***	3 < 1 and 2* 1 and 3 < 2***	1 < 2 and 3***

^*^p < 0.05; ^***^p < 0.001. Effect sizes are given as eta-squared values.

Cluster 1 (*n* = 50) was named *Lonely*, as it included patients who reported the lowest scores on social support. This cluster also showed the lowest scores on resilience, and scores around average on task-oriented coping and positive affect. Cluster 2 (*n* = 50) was named *Low Risk* since it included patients who scored the highest on resilience, task-oriented coping, positive affect and social support. Cluster 3 (*n* = 54) was named *Avoidant,* since it included patients with the lowest scores on task-oriented coping and positive affect, and with average scores in resilience and social support.

**Figure 1:**
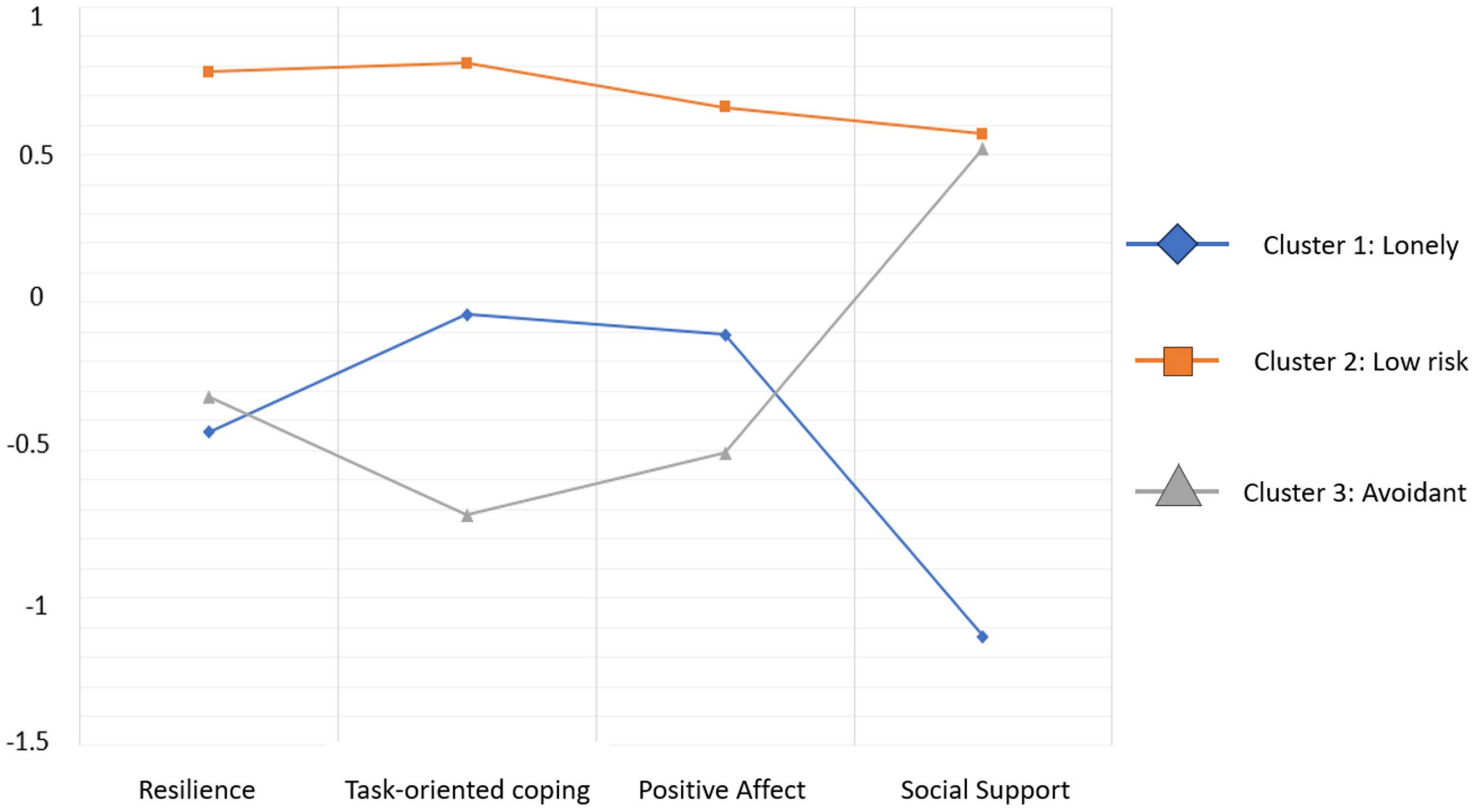
Standardized mean scores of the identified clusters in the variables of interest.

Figure 1 shows the standardized mean Z scores of the clusters considered. As it can be seen in Figure 1, cluster 2 may represent a more positive or adaptive cluster compared with clusters 1 and 3.

### 3.2 Cluster differences in depressive symptoms at follow-up assessments

Significant differences in the Beck Depression Inventory scores were found between clusters 3 months after MI (*F* = 5.10; *p* < 0.01; effect size = 0.27) and 12 months after MI (*F* = 7.56; *p* > 0.01; effect size = 0.37).

Specifically, 3 months after MI, participants from the *Low Risk* (cluster 2) group showed significantly lower depressive symptoms than those from the *Lonely* (cluster 1) and *Avoidant* (cluster 2) groups. This difference remained statistically significant 12 months after MI (see Table 3). No statistically significant differences in depressive symptoms were found at 3-month or at 12-month follow up between the *Lonely* (cluster 1) and *Avoidant* (cluster 2) profiles.

**Table 3:** Means, standard deviations and differences in depressive symptoms and each PTSS subscale at 3-month and 12-month follow-up by cluster group.

Cluster	*N*	Mean (SD)	Lower than	Higher than
**BDI scores at 3-month follow-up**				
1. Lonely	50	6.41 (4.69)		2*
2. Low risk	50	3.49 (3.65)	1* 3*	
3. Avoidant	54	6.46 (6.17)		2*
**BDI scores at 12-month follow-up**				
1. Lonely	31	7.28 (4.28)		2**
2. Low risk	34	3.03 (3.18)	1** 3*	
3. Avoidant	39	5.70 (5.28)		2*
**CAPS scores at 3-month follow-up**				
1. Lonely	50	12.44 (9.80)		2*
2. Low risk	50	6.94 (8.19)	1* 3*	
3. Avoidant	54	12.39 (12.23)		2*
**Re-experiencing at 3-month follow-up**				
1. Lonely	50	3,04 (4.13)		
2. Low risk	50	2.16 (4.07)		
3. Avoidant	54	2.98 (4.19)		
**Avoidance at 3-month follow-up**				
1. Lonely	50	3.18 (3.62)		
2. Low risk	50	1.36 (2.13)	3**	
3. Avoidant	54	4.17 (5.84)		2**
**Hyperarousal at 3-month follow-up**				
1. Lonely	50	6.18 (4.74)		2**
2. Low risk	50	3.38 (3.39)	1**	
3. Avoidant	54	5.24 (4.48)		
**CAPS scores at 12-month follow-up**				
1. Lonely	31	10.80 (1.94)		2**
2. Low risk	34	5.82 (1.00)		
3. Avoidant	39	8.07 (1.29)		
**Re-experiencing at 12-month follow-up**				
1. Lonely	31	3.68 (4.37)	2** 3**	
2. Low risk	34	1.38 (2.50)		1**
3. Avoidant	39	1.38 (1.77)		1**
**Avoidance at 12-month follow-up**				
1. Lonely	31	4.06 (4.97)	2*	
2. Low risk	34	1.41 (1.96)		1*
3. Avoidant	39	3.44 (4.41)		
**Hyperarousal at 12-month follow-up**				
1. Lonely	31	5.26 (3.65)		
2. Low risk	34	3.62 (3.76)		
3. Avoidant	39	4.33 (3.83)		

*p < 0.05; **p < 0.01; ***p < 0.001.

### 3.3 Cluster differences in PTSS at follow-up assessments

Significant differences in MI-induced PTSS were found between clusters both 3 months after MI (*F* = 4.78; *p* < 0.05; effect size = 0.21 see Table 3) and 12 months after MI (*F* = 5.57; *p* < 0.01; effect size = 0.23).

Specifically, 3 months after MI, participants with the *Low Risk* (cluster 2) profile scored significantly lower in PTSS than participants from the *Lonely* (cluster 1) and *Avoidant* (cluster 3) groups. At 12-month follow-up, *Low Risk* (cluster 2) participants scored significantly lower in PTSS than participants with the *Lonely* (cluster 1) profile. No statistically significant differences were found between participants pertaining to the *Low Risk* (cluster 2) and *Avoidant* (cluster 1) profiles 12 months after MI.

### 3.4 Secondary analyses: differences in each PTSD subscale

Three months after MI, statistically significant differences between clusters were shown for the CAPS subscales avoidance (*F* = 4.80; *p* < 0.05; effect size = 0.27) and hyperarousal (*F* = 5.63; *p* < 0.01; effect size = 0.27), but not for re-experiencing. 12 months after MI, statistically significant differences between clusters were shown for re-experiencing (*F* = 6.44; *p* < 0.01; effect size = 0.34) and avoidance (*F* = 4.03; *p* < 0.05; effect size = 0.27), but not for hyperarousal.

### 3.5 Factor analysis and demographic differences between groups

The results obtained from the exploratory factor analysis of the psychosocial variables evaluated (resilience, task-oriented coping, positive affect, and social support) suggested the existence of one factor (KMO and Bartlett’s test: 0.57, *p* < 0.00). Thus, variables would all load in a one-factor structure. Results from demographic differences between groups can be found in Supplementary Tables S1, S2. The factor loadings of each variable on the factor are in all cases greater than 0.70. No statistically significant differences were found in any of the demographic or medical variables, suggesting that participants characteristics were similar in all three profiles.

## 4 Discussion

We found three clusters, which were a *Lonely* cluster with patients scoring low in social support, and resilience and an average level of task-oriented coping and positive affect; a *Low-Risk* cluster with patients scoring high in all four positive psychology variables, namely, resilience, task-oriented coping, positive affect, and social support; and an *Avoidant* cluster with patients scoring low on task-oriented coping and positive affect and an average level of resilience and social support. These clusters showed statistically significant differences in depressive symptoms and PTSS both at 3-month and 12-month follow-up assessments. This might be because difficulties in experiencing positive emotions are associated with depression and PTSD and might reflect a reaction to the traumatic event, which changes over time (Vanderlind et al., 2020; Wolkenstein et al., 2022). No cluster differences were found in any other demographic or medical variables, suggesting that participants characteristics were similar, and that differences in depressive symptoms and PTSS could be attributed in differences in resilience, coping, positive affect and social support. Additionally, factor analysis revealed that all three positive psychosocial variables showed a better fit to a one-factor structure. These results suggest that both internal (e.g., resilience) and external (i.e., social support) patient’s resources can be considered at the same time. This aligns with previous research that also considered both social factors and individual differences when it comes to face and overcome potentially traumatic events (Lee et al., 2013; Martínez-Martí and Ruch, 2017). We detected adaptive and maladaptive profiles for depressive symptoms and PTSS secondary to acute MI. In contrast to the maladaptive profiles *Lonely* and *Avoidant*, the adaptive profile, characterized as *Low-Risk*, showed the lowest level of depressive symptoms and PTSS at 3- and 12-month post-MI. This is in line with previous findings showing that social support and resilience are associated with reduced depressive symptoms (Leifheit-Limson et al., 2010; Pfeiffer et al., 2011; Arabadjian et al., 2023) and PTSS (Dinenberg et al., 2014; Kirchner et al., 2022) in cardiac patients. Lack of social support has been related to increased morbidity and mortality and increased cardiac risk after acute MI (Barth et al., 2010). In addition, evidence indicates that greater social support is associated with improved self-care and overall quality of life in heart failure patients (Dunbar et al., 2005, 2008; Khaledi et al., 2015). Moreover, social support is highly associated with resilience (Stewart and Yuen, 2011) and resilient patients were shown to be at lower risk of developing acute stress disorder and experience PTSS (Meister et al., 2015). Further studies have shown that resilience is linked to increased compliance with treatment recommendations, improved health-related quality of life, decreased severity of pain, adherence to exercise routines and better physical outcomes like decreased HbA1c levels (Stewart and Yuen, 2011). Moreover, resilient patients reported personal growth, rather than depressive symptoms and PTSS, when confronted with physical illness or traumatic events (Stewart and Yuen, 2011).

Our analyses showed that participants scoring higher in task-oriented coping (*Low-Risk* cluster) also showed lower levels of depressive symptoms and PTSS. According to Lazarus and Folkman (1984), coping strategies can be divided into emotion-focused and task-oriented coping. Emotion-focused coping strategies seems to be positively associated with depressive symptoms (Klein et al., 2007) and PTSS (Chung et al., 2008; Ayers et al., 2009) in patients with cardiovascular disease. This may be due to the fact that individuals with emotion-focused coping may be overwhelmed by feelings that cannot be regulated in situations out of personal control like acute MI. Our finding is in line with a previous study, reporting a negative association between task-oriented coping and depressive symptoms in patients following MI (Messerli-Bürgy et al., 2015). Moreover, task-oriented coping predicted reduced risk of major adverse cardiac events over a follow-up period of 5 years in patients with a previous MI (Messerli-Bürgy et al., 2015); therefore, task-oriented coping seems to be beneficial if the “problem” can be solved actively. Further studies should address therapeutic approaches to improve self-efficacy and active problem-solving strategies.

Positive affect seems to have an influence on depressive symptoms. In a prospective observational clinical study, optimism predicted reduced depressive symptoms 12 months after MI, independent of demographic and clinical factors (Ronaldson et al., 2015). Moreover, increased positive affect was associated with a reduced risk of 10-year incidence of CHD (Davidson et al., 2010) and reduced mortality risk in patients with cardiovascular disease (Brummett et al., 2005; Hoen et al., 2013). Further beneficial effects of positive affect have been shown in terms of better sleep, increased physical activity, medication adherence (Sin et al., 2015; Ong et al., 2017), and inflammation (Zuccarella-Hackl et al., 2023). The valuable effect of positive affect on PTSS is in line with previous research showing that positive psychosocial factors prevent the development of PTSS after exposure to a traumatic event (McCanlies et al., 2014). This, because individuals who experience positive affect more frequently and intensely might be better able to recover from negative emotional experiences (Tugade and Fredrickson, 2004). According to the Model by Kubzansky et al. (2018), positive affect may contribute to the development of resilience, social support, and task-oriented coping strategies. This psychosocial pathway and stress-buffering effect may prevent the development of depressive symptoms and PTSS following MI.

This study also has some clinical implications. Firstly, patients with a maladaptive profile of positive psychosocial factors could be identified to prevent potentially negative clinical outcomes, in particular depressive symptoms and PTSS. Also, focusing on enhancing patients’ resources could also improve patient’s ability to adapt to illness and other adversities (Aspinwall et al., 2010). Previous studies have found a relationship between positive psychological variables and cardiovascular health (Boehm and Kubzansky, 2012), which could also be extended to MI patients. Gaining an understanding of how positive psychosocial factors contribute to preventing negative emotions like depressive symptoms and PTSS in clinical population can facilitate the development of more effective treatments and provide personalized approaches that align with individual patient profiles (Windgassen et al., 2018). Individual-level interventions, such as mindfulness-based programs and positive psychological interventions may potentially increase psychological well-being and decrease patients’ distress (Kubzansky et al., 2018). Also, cluster analysis and identification of patient profiles could help health professionals to identify patients’ needs and strengths, which enables the design of interventions tailored to the unique dispositions and risks of targeted groups (Doron et al., 2015).

Our study has notable limitations: We included highly distressed patients following acute MI participating in a RCT aimed at preventing PTSS caused by a cardiac event. Therefore, our findings cannot be generalized to other patient populations and populations of MI patients in general. However, it must be mentioned that this homogenous sub-population is at increased risk for developing PTSD and it might be important to focus on this group.

The present study does not allow to infer the direction of causality. We measured positive psychosocial variables at one time point only, which cannot capture their potential temporal dynamics. Also, our study did not take into account baseline measures of depression or pre-existent PTSS due to different causes. In addition, scores in variables such as positive affect may fluctuate over time. Besides, social support was only measured once at hospital admission, and the variables resilience, task-oriented coping and positive affect were only measured at the 3-month follow-up interview. This methodology aimed to reduce the burden on patients and the length of the clinical interview. However, this could lead to the assumption that participant’s scores on these variables do not change over time. Future studies should include repeated measures of all the assessed variables to examine their stability over time and properly address temporal relationships between variables. Therefore, longitudinal studies of positive psychosocial factors in relation to depressive symptoms and PTSS are needed.

## 5 Conclusion

The purpose of this study was to examine the association of clusters of positive psychosocial factors with both MI-induced PTSS and depressive symptoms, independent of demographic factors. Three distinct clusters emerged from the analysis: (1) the “lonely cluster” characterized by the lowest levels of social support, average task-oriented coping, and positive affect; (2) the “low risk cluster” demonstrating the highest levels of resilience, task-oriented coping, positive affect, and social support; and (3) the “avoidant cluster” exhibiting the lowest levels of task-oriented coping, positive affect, average resilience, and social support. Furthermore, the study shows, that positive psychosocial factors may influence the development of depressive symptoms and PTSS after a MI. Future study may want to investigate whether interventions to increase positive psychosocial factors may potentially reduce depressive symptoms and PTSS in patients after acute MI as well as all-cause and cardiovascular disease mortality.

## Data availability statement

The raw data supporting the conclusions of this article will be made available by the authors, without undue reservation.

## Ethics statement

The studies involving humans were approved by State of Bern’s ethics committee. The studies were conducted in accordance with the local legislation and institutional requirements. The participants provided their written informed consent to participate in this study.

## Author contributions

CZ-H: Writing – original draft, Writing – review & editing. LJ-G: Formal analysis, Methodology, Writing – original draft, Writing – review & editing. RvK: Conceptualization, Writing – review & editing. MP: Writing – review & editing. LJ: Writing – review & editing. RL-M: Writing – review & editing. HZ: Conceptualization, Writing – review & editing. J-PS: Conceptualization, Writing – review & editing. JB: Conceptualization, Writing – review & editing. US: Conceptualization, Writing – review & editing. KL: Writing – review & editing.
