# Supplementary material for: Positive psychosocial factors and the development of symptoms of depression and posttraumatic stress symptoms following acute myocardial infarction
Source: Front Psychol. 2023 Dec 4;14:1302699. doi: 10.3389/fpsyg.2023.1302699 (PMC10725949; doi:10.3389/fpsyg.2023.1302699)
Supplement: Supplementary file 1 [file Data_Sheet_1.docx]

Supplementary Material

# Supplementary Figure


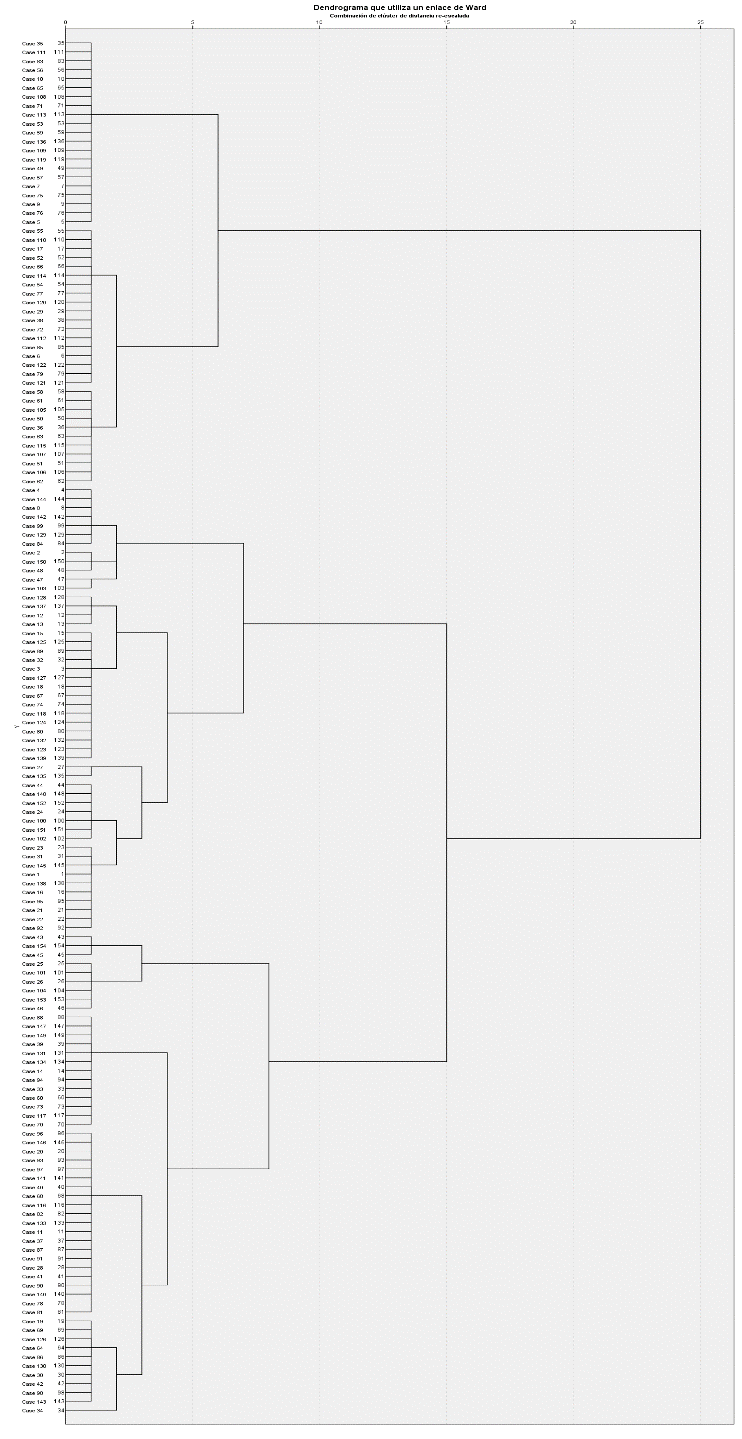


**Supplementary Figure 1.** Cluster dendogram using the Ward method. Dashed line identifies the 3-cluster solution.

| Supplementary Table 1.  Chi-square test for cluster differences in demographic and medical variables. | | | |
| --- | --- | --- | --- |
|  | Chi-square value | Df | p |
| Intervention group | 0.64 | 2 | 0.73 |
| Gender, male | 4.23 | 2 | 0.11 |
| ST elevation MI | 4.12 | 2 | 0.13 |
| Recurrent MI | 2.05 | 2 | 0.36 |
| Hypertension | 1.22 | 2 | 0.55 |
| Hypercholesterolemia | 1.79 | 2 | 0.41 |
| Diabetes | 3.85 | 2 | 0.15 |

MI, myocardial infarction

| Supplementary Table 2.  One-way ANOVA for cluster differences in demographic and medical variables. | | |
| --- | --- | --- |
|  | F value | p |
| Age | 0.34 | 0.72 |
| Body mass index | 0.43 | 0.65 |
| Systolic blood pressure | 0.58 | 0.56 |
| Diastolic blood pressure | 0.68 | 0.51 |

**
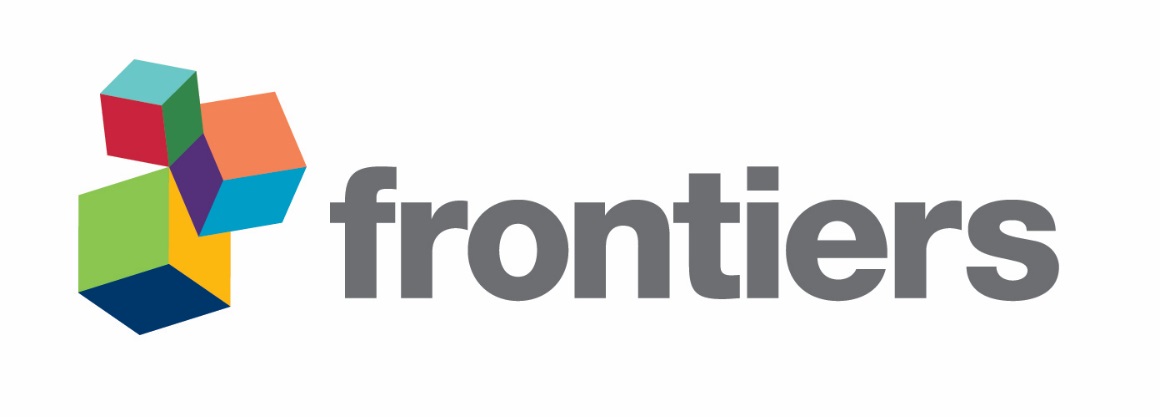
**
